# Metabolomics uncovers the diabetes metabolic network: from pathophysiological mechanisms to clinical applications

**DOI:** 10.3389/fendo.2025.1624878

**Published:** 2025-09-04

**Authors:** Zijie Xu, Yujia Zhou, Ruijie Xie, Zhongxing Ning

**Affiliations:** ^1^ Guangxi Hospital Division of The First Affiliated Hospital, Sun Yat-sen University, Nanning, China; ^2^ Department of Infectious Diseases and Public Health, City University of Hong Kong, Hong Kong, Hong Kong SAR, China; ^3^ Faculty of Medicine, Heidelberg University, Heidelberg, Germany; ^4^ Sun Yat-sen University, Guangzhou, China; ^5^ Department of Hypertension and Vascular Disease, The First Affiliated Hospital of Sun Yat-sen University, Guangzhou, China

**Keywords:** metabolomics, diabetes mellitus, metabolic reprogramming, biomarkers, clinical translation, precision medicine

## Abstract

Diabetes mellitus (DM) represents a complex metabolic disorder posing urgent diagnostic and therapeutic challenges worldwide. Traditional biomarkers such as HbA1c and OGTT fail to capture the dynamic nature of metabolic remodeling underlying DM pathophysiology. Metabolomics, by offering real-time, systems-level insights into small-molecule dynamics, has emerged as a promising strategy for both early disease detection and therapeutic target discovery. Recent studies have highlighted the diagnostic and prognostic value of metabolites, including branched-chain amino acids, lipid derivatives, and bile acids. Despite its immense potential, the clinical application of metabolomics remains hindered by technical limitations, such as cross-cohort standardization and data interpretation complexity. Future advances integrating artificial intelligence and multi-omics strategies may transform metabolomics from an exploratory tool to a clinical mainstay in diabetes management. This review offers a comprehensive synthesis of recent advances in metabolomics-driven diabetes research, with a particular focus on elucidating key metabolic pathways, identifying emerging biomarkers, and exploring translational opportunities. To fully realize the clinical potential of metabolomics, further efforts toward analytical standardization, cross-cohort validation, and the integration of artificial intelligence–powered tools will be essential to bridge the gap from bench to bedside in diabetes care.

## 1 Introduction

Diabetes mellitus (DM) is an escalating global health crisis, currently affecting over 537 million people worldwide, with projections indicating an increase to 643 million by 2030 and 783 million by 2045, according to the International Diabetes Federation (1). As a leading cause of morbidity and mortality, DM imposes a substantial socioeconomic burden due to its diverse complications, including cardiovascular disease, nephropathy, neuropathy, and retinopathy (2). Despite notable advances in diagnostics and treatment, the global prevalence of diabetes continues to rise, underscoring the urgent need for more effective and personalized disease management strategies (3).

Despite the widespread use of traditional diagnostic markers such as hemoglobin A1c (HbA1c), the oral glucose tolerance test (OGTT), and fasting plasma glucose (FPG), these methods have inherent limitations in capturing the dynamic and multifactorial nature of diabetes pathogenesis. HbA1c levels, for instance, are influenced by variations in erythrocyte lifespan, potentially leading to inaccuracies in individuals with anemia or hemoglobinopathies (4). Similarly, although OGTT is the gold standard for diabetes diagnosis, it reflects only a single time point of glucose metabolism and fails to account for fluctuations in insulin sensitivity and metabolic adaptations (5). FPG remains widely used due to its accessibility, low cost, and standardized protocols (6). However, it has several inherent limitations: requiring prolonged fasting (≥8 hours), representing only a single metabolic snapshot, and exhibiting sample stability concerns (7). Furthermore, while genome-wide association studies (GWAS) have identified numerous genetic loci associated with diabetes susceptibility, genetic predisposition alone cannot fully explain the disease’s heterogeneity (8).

In diabetes treatment, the molecular mechanisms underlying β-cell dysfunction and tissue-specific insulin resistance remain incompletely understood, hindering the development of targeted therapies (9). Furthermore, a subset of patients demonstrates suboptimal responses to first-line treatments such as metformin, highlighting the need for personalized therapeutic strategies (10). The absence of reliable early-stage biomarkers and individualized treatment targets underscores the urgent need for innovative approaches capable of comprehensively mapping metabolic perturbations in diabetes.

Metabolomics is the large-scale study of small molecules within biological systems such as cells, biofluids, tissues, or entire organisms (11). As a rapidly evolving discipline following genomics and proteomics, metabolomics provides a comprehensive snapshot of an organism’s metabolic composition (12). Since the launch of the Human Metabolome Database (HMDB) in 2007, metabolomics has advanced significantly and has been widely applied across multiple domains, including disease diagnostics, drug discovery, nutritional science, toxicology, environmental research, and plant biology (13–17). Analytical platforms such as mass spectrometry (MS), nuclear magnetic resonance (NMR), and various chromatography methods (e.g., HPLC, GC) enable precise quantification of thousands of metabolites (11). Recent innovations, such as spatial and single-cell metabolomics, are further enhancing its potential in precision medicine (18, 19). Compared with other omics technologies, metabolomics offers distinct translational advantages. While genomics and transcriptomics capture static or upstream regulatory information, and proteomics is limited by protein coverage and complexity, metabolomics reflects the integrated outcomes of biological processes and environmental exposures (20). This capacity to detect real-time functional changes makes metabolomics particularly valuable for monitoring disease states, identifying therapeutic targets, and enabling individualized interventions in diabetes.

One of the most powerful applications of metabolomics in diabetes lies in its capacity to decode metabolic reprogramming, a hallmark of disease pathogenesis marked by disrupted nutrient sensing, energy metabolism, and hormonal signaling (21). At the tissue level, skeletal muscle in individuals with diabetes exhibits mitochondrial dysfunction, shifting from efficient oxidative phosphorylation to incomplete fatty acid oxidation and the accumulation of lipotoxic intermediates, such as long-chain acylcarnitine, which impair insulin signaling (22). Concurrently, inflamed and insulin-resistant adipose tissue secretes pro-inflammatory cytokines that exacerbate hepatic gluconeogenesis and contribute to systemic insulin resistance (23). These systemic perturbations ultimately lead to pancreatic β-cell dedifferentiation, wherein chronic hyperglycemia induces epigenetic silencing of key metabolic regulators (e.g., PDX1 and MAFA). This blunts glucose-stimulated insulin secretion, perpetuating a vicious cycle of metabolic deterioration. Through high-resolution metabolic profiling, metabolomics enables the detection of these maladaptive changes, offering new opportunities for early diagnosis, patient stratification, and development of targeted therapeutics tailored to specific metabolic subtypes of diabetes.

However, significant challenges remain in translating metabolomic findings into clinical practice, including the standardization of analytical protocols, cross-population validation, and the biological interpretation of complex datasets. Consequently, a comprehensive overview of the role of metabolomics in decoding the metabolic network of diabetes is urgently needed. This review therefore aims to (1) summarize recent advances in metabolomics technologies relevant to diabetes, (2) elucidate key mechanisms of metabolic reprogramming, (3) highlight promising biomarkers and patient stratification strategies, and (4) discuss translational applications and future challenges. Through this synthesis, we aim to delineate the potential of metabolomics in advancing precision medicine in diabetes management.

## 2 Metabolomics technologies

### 2.1 Analytical platforms

Metabolomics has emerged as a foundational discipline in contemporary biomedical research, offering a systems-level approach to characterize metabolic perturbations in complex diseases such as diabetes. The field employs diverse analytical platforms, each with distinct advantages and technical limitations.

Mass spectrometry (MS) is regarded as the gold standard in metabolomic investigations, owing to its exceptional sensitivity, mass resolution, and metabolite coverage (**Table 1**). Current MS-based metabolomics primarily employs two complementary strategies: untargeted and targeted approaches (12). Untargeted metabolomics relies on high-resolution mass spectrometers (HRMS), including Fourier transform ion cyclotron resonance (FT-ICR), time-of-flight (TOF), and Orbitrap instruments, to achieve comprehensive metabolic profiling (24, 25). These platforms offer sub-ppm mass accuracy, enabling confident metabolite identification. For example, a UHPLC-Q Exactive HF-X MS-based platform provides sub-ppm mass accuracy (± 10 ppm) and can detect over 2,000 metabolite ions in untargeted metabolomics, highlighting its superior performance in high-throughput metabolite profiling (26). In contrast, targeted metabolomics focuses on the accurate quantification of predefined metabolites or pathways, typically employing triple quadrupole (QQQ) mass spectrometers operated in multiple reaction monitoring (MRM) mode to enhance sensitivity and specificity (24, 27).

**Table 1:** Comparison of metabolomics technologies.

Technology	Detection Principle	Main Advantages	Main Disadvantages	Applicable Scenarios
NMR	Nuclear magnetic resonance	Non-destructive, high reproducibility, quantitative	Low sensitivity, limited to specific metabolites	Biofluid metabolomics, metabolic pathway studies
MS	Mass spectrometry ion detection	High sensitivity, capable of detecting low-abundance metabolites	Requires complex sample preparation	Large-scale metabolomic screening
GC-MS	Gas chromatography-mass spectrometry	Suitable for volatile metabolites	Requires derivatization	Small molecule analysis, such as fatty acids and sugars
LC-MS	Liquid chromatography-mass spectrometry	Suitable for both polar and non-polar metabolites	Complex data processing is required	Drug metabolism, lipidomics
CE-MS	Capillary electrophoresis-mass spectrometry	High resolution, ideal for charged small molecules	Influenced by buffer composition, lower reproducibility	Neuro-metabolism, energy metabolism research
FT-ICR-MS	Fourier transform ion cyclotron resonance mass spectrometry	Ultra-high resolution, precise mass accuracy	Expensive instrumentation, high maintenance cost	Lipidomics, environmental metabolomics
IMS-MS	Ion mobility spectrometry-mass spectrometry	Separates structural isomers, improves metabolite identification	Requires advanced data interpretation	Lipidomics, biomarker discovery
MALDI-MSI	Matrix-assisted laser desorption/ionization mass spectrometry imaging	High spatial resolution, direct tissue imaging	Requires specialized sample preparation	Cancer metabolomics, neurodegenerative disease studies
Raman Spectroscopy	Light scattering-based metabolite detection	Non-invasive, label-free, real-time analysis	Lower sensitivity compared to MS	Single-cell metabolism, live-cell monitoring

The coupling of MS with liquid chromatography (LC) or gas chromatography (GC) significantly improves metabolite separation and identification. LC-MS is particularly well-suited for analyzing both polar and non-polar metabolites, whereas GC-MS is primarily employed for volatile and thermally stable compounds. LC-MS demonstrates exceptional versatility in separating a wide range of compound classes across a broad polarity spectrum (28). This technique achieves a mass accuracy of 5–10 ppm in quantifying polar metabolites such as branched-chain amino acids, enabling the precise identification of type 2 diabetes mellitus (T2DM) progression biomarkers in large-scale cohort studies like the Framingham Heart Study (29). The predominant use of soft-ionization techniques in LC-MS facilitates molecular ion formation with minimal fragmentation, thereby enhancing the identification of unknown compounds (30). A key advantage of LC-MS over GC-MS is that it typically eliminates the need for chemical derivatization, as it does not require high temperatures or compound volatility. Additionally, LC-MS offers superior sensitivity across a broad molecular weight range (from <600 Da to larger molecules), including phospholipids, conjugated bile acids, glycosides, and sugars (31). GC-MS remains the gold standard for analyzing volatile and thermally stable metabolites, is capable of separating naturally volatile compounds (e.g., ketones, aldehydes) as well as non-volatile compounds rendered volatile through derivatization (e.g., sugars, amino acids) (32). This technique shows particular efficacy in fatty acid analysis and has proven invaluable for clinical research on metabolic disorders (33). However, GC–MS is limited by relatively poor reproducibility, due to potential structural alterations during derivatization (34).

Nuclear magnetic resonance (NMR) spectroscopy is another cornerstone technology in metabolomics, offering non-destructive, highly reproducible, and quantitative analysis of metabolites. NMR is particularly well-suited for studying complex biofluids and tissues, as it requires minimal sample preparation while offering detailed structural insights into metabolites. Additionally, NMR can be applied to *in vivo* tissues and living samples, enabling real-time metabolic profiling and dynamic flux analysis (35). NMR-based metabolomics has proven valuable in identifying metabolic signatures associated with diabetes progression and complications. For example, it has revealed dysregulation of branched-chain amino acids (BCAAs) and lipid metabolism in patients with T2DM (29). However, NMR’s relatively lower sensitivity compared to MS limits its ability to detect low-abundance metabolites. Recent advancements in high-resolution two-dimensional NMR spectroscopy may help overcome this limitation and broaden the applicability of NMR in metabolomics research (36).

In recent years, metabolomics technology has been continuously developing. In addition to traditional NMR and MS analysis methods, a variety of emerging technologies have shown unique advantages in the detection and quantification of specific metabolites. Among them, capillary electrophoresis-mass spectrometry (CE-MS) combines the high resolution of capillary electrophoresis with the high sensitivity of mass spectrometry (37). It is particularly suitable for the quantitative analysis of small molecule polar metabolites (such as organic acids, nucleotides, and amino acids) and has been widely used in the study of neural metabolism and energy metabolism. Fourier transform ion cyclotron resonance mass spectrometry (FT-ICR-MS) has unique advantages in the accurate identification of complex metabolites with its ultra-high resolution and extremely low mass error, especially in lipidomic and environmental metabolomics research (38). On the other hand, ion mobility spectrometry-mass spectrometry (IMS-MS) can distinguish metabolites with similar structures (such as isomers) by combining gas phase ion separation and mass spectrometry analysis, and has important value in lipidomic and biomarker screening research (39). This innovation has been particularly impactful in lipidomics, as evidenced by the successful resolution of ceramide isomers (e.g., C26:0) in clinical studies, thereby improving lipid profiling accuracy in metabolic diseases (40). Notably, matrix-assisted laser desorption/ionization mass spectrometry imaging (MALDI-MSI) is a high-spatial-resolution metabolomics imaging technique that can directly detect metabolite distribution on tissue sections (41). For example, MALDI-MSI has been used for spatial imaging of the metabolic profile of brain tissue in patients with Alzheimer’s disease, revealing changes in key metabolic pathways during disease progression (42). As reported by Prentice et al., MALDI-MSI has successfully mapped the *in situ* spatial distribution of hormones such as insulin, glucagon, and their protein variants in human and mouse islets with high spatial resolution. Their study revealed distinct hormone distribution patterns between the core region (β cells) and the peripheral region (α cells) of the islets, confirmed through serial section co-localization immunofluorescence and mass spectrometry imaging (43). In addition, Raman spectroscopy, as a non-invasive, label-free metabolomics detection method, has been gradually applied to *in vivo* single-cell metabolic monitoring and cancer diagnosis (44). Combined with surface-enhanced Raman scattering (SERS) technology, this method can significantly improve the sensitivity of metabolite detection and show great potential in the fields of stem cell metabolism and personalized medicine (45). Nevertheless, MS-based metabolomics continues to face challenges, including data complexity, metabolite identification difficulties, and the requirement for advanced bioinformatics solutions.

The continuous development of these technologies has not only broadened the scope of metabolite detection but also improved the accuracy of metabolomics data analysis. In the future, the combined application of multiple technologies will become an important trend in metabolomics research, providing more powerful technical support for precision medicine, disease mechanism research, and biomarker discovery.

### 2.2 Data analysis workflow in metabolomics

**Figure 1:**
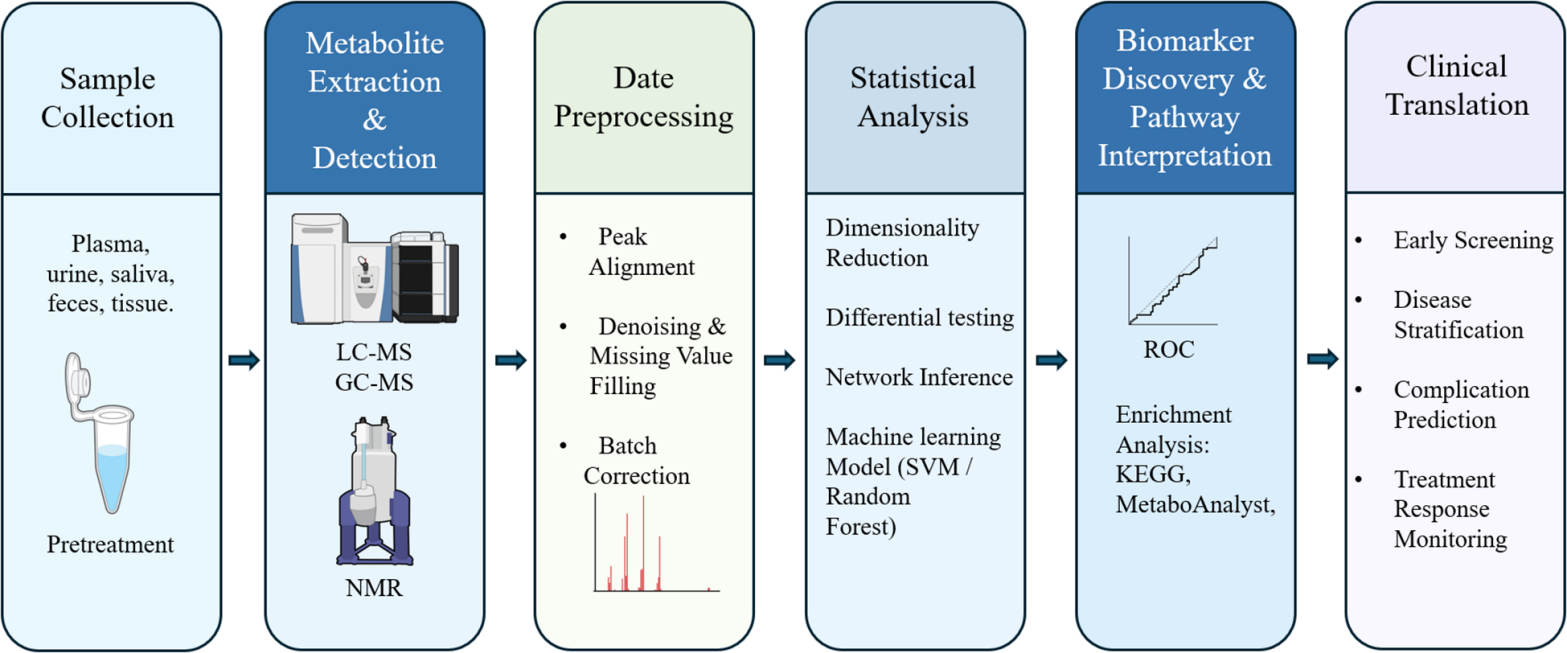
Integrated workflow of metabolomics application in diabetes. The six-step pipeline starts with (1) sample collection and pretreatment from various biological fluids or tissues (e.g., plasma, urine, feces); (2) metabolite extraction and detection using LC-MS, GC-MS, or NMR platforms; (3) data preprocessing, including peak alignment, denoising, and batch correction; (4) statistical analysis including dimensionality reduction, differential testing, network inference, and machine learning models (e.g., SVM, random forest); (5) biomarker discovery and pathway interpretation, guided by ROC curve analysis and enrichment tools such as KEGG and MetaboAnalyst; and (6) clinical translation, encompassing early screening, disease stratification, prediction of complications, and treatment response monitoring.

Metabolomics data analysis is a complex, multi-stage process that demands a methodical and rigorous approach to convert raw spectral data into biologically interpretable results. A critical step after sample acquisition is data preprocessing, including noise reduction, peak detection, and spectral alignment (**Figure 1**), typically performed using specialized software such as MS-DIAL and XCMS (46). Noise reduction filters out random signal fluctuations, while peak detection and alignment standardize data across samples to ensure reproducibility. Normalization is then applied to minimize technical variability (e.g., batch effects) and improve cross-dataset comparability (47). Subsequently, an exploratory data assessment is conducted using visualization tools (e.g., histograms, boxplots) to detect outliers and validate data quality before further statistical analysis.

Metabolomics data analysis can be broadly classified into hypothesis-driven and hypothesis-free approaches. In hypothesis-driven analyses, parametric (e.g., Student’s t-test) or non-parametric tests (e.g., Mann–Whitney U test) are applied to compare metabolite concentrations between defined groups—such as diabetic patients versus healthy controls—depending on data distribution (48). For hypothesis-free (exploratory) analyses, multivariate statistical methods are employed to reveal hidden metabolic patterns. Principal Component Analysis (PCA), an unsupervised approach, reduces data dimensionality while highlighting major sources of metabolic variation (49). Supervised methods, including Partial Least Squares Discriminant Analysis (PLS-DA) and Orthogonal Partial Least Squares (OPLS), further refine interpretations by suppressing noise and maximizing group separation (50). These are frequently integrated with PCA to isolate biologically relevant metabolic changes from confounding variation (51).

Accurate metabolite annotation and identification are critical for deriving biological insights from metabolomics data. This process entails matching experimental spectral features against curated reference databases (e.g., METLIN), which house extensive collections of standardized metabolite spectra (52). For uncharacterized or novel compounds, advanced computational pipelines such as SIRIUS coupled with CSI: FingerID enable *de novo* structural elucidation by analyzing mass spectral patterns and fragmentation signatures (53). This dual-strategy framework facilitates comprehensive metabolite characterization, bridging the gap between known biochemical entities and novel metabolic discoveries, thereby significantly enhancing the depth of metabolomic investigations.

To elucidate the biological relevance of identified metabolites, systematic pathway analysis is conducted to integrate metabolites into established metabolic networks. Widely utilized platforms such as MetaboAnalyst and the Kyoto Encyclopedia of Genes and Genomes (KEGG) database facilitate this process (54, 55). MetaboAnalyst provides an intuitive interface for metabolite set enrichment analysis and pathway visualization, whereas KEGG offers curated, species-specific metabolic pathway references. Through this functional mapping, researchers can pinpoint dysregulated metabolic pathways associated with specific conditions, yielding mechanistic insights into disease pathogenesis and revealing potential intervention targets (56).

The integration of machine learning (ML) in metabolomics has transformed the field by facilitating high-dimensional pattern recognition and the development of predictive metabolic models. Advanced algorithms, including random forest and support vector machines (SVM), are routinely employed for feature selection and classification model construction (57). For instance, a large-scale prospective cohort study leveraging NMR-based metabolomics combined with ML approaches identified a nine-metabolite panel that significantly enhanced the prediction of T2DM progression in prediabetic individuals (58). A primary goal of metabolomics research is the discovery and validation of biomarkers. The diagnostic potential of candidate biomarkers is rigorously assessed through receiver operating characteristic (ROC) curve analysis, with the area under the curve (AUC) serving as a key metric for predictive performance evaluation (59). Promising biomarkers undergo multi-stage validation, including *in vivo* studies using animal models or independent clinical cohorts, to verify their biological relevance and translational applicability (60, 61).

To elucidate the complexity of biological systems, integrated multi-omics approaches have emerged as a powerful strategy. The synergistic combination of metabolomics with complementary omics layers (e.g., transcriptomics, proteomics) enables systematic reconstruction of molecular interactions and regulatory networks (62). Advanced computational frameworks, including Multi-Omics Factor Analysis (MOFA) and Weighted Gene Co-Expression Network Analysis (WGCNA), provide robust solutions for data integration and biological interpretation, offering holistic insights into disease pathogenesis and potential intervention strategies (63, 64). This systems biology paradigm significantly enhances the discovery of mechanistic biomarkers and druggable targets, advancing both basic research and translational applications.

## 3 Metabolic reprogramming and biomarker discovery

The pathogenesis of diabetes involves complex metabolic reprogramming, characterized by eventual insulin resistance and β-cell dysfunction (65). Insulin resistance, a defining feature of T2DM, is strongly associated with impaired mitochondrial function in skeletal muscle (66). β-cell dysfunction represents a fundamental pathological mechanism common to both T1DM and T2DM, characterized by progressive insulin secretory failure and consequent hyperglycemia (67, 68). Understanding the abnormalities of key metabolic pathways in the development of diabetes and making timely early diagnosis are crucial for mitigating disease progression and preventing complications (69).

### 3.1 Amino acid metabolites

**Figure 2:**
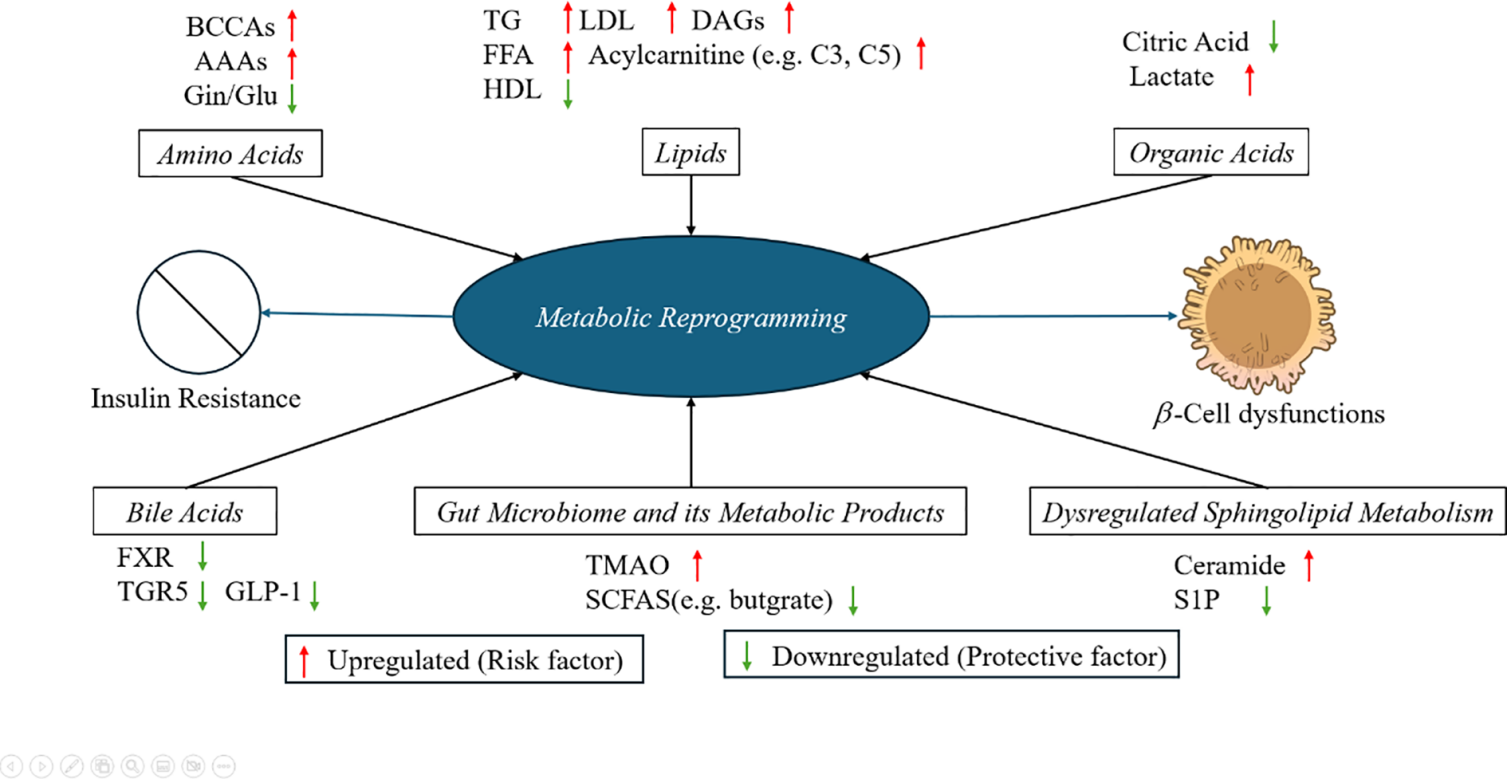
Visual summary of metabolic reprogramming in diabetes. This schematic illustrates six key metabolic subsystems that undergo characteristic shifts during the development of diabetes: amino acids, lipids, organic acids, bile acids, gut microbiota–derived metabolites, and sphingolipids. These metabolic alterations contribute to two central pathological processes: insulin resistance and β-cell dysfunction. Red arrows (↑) indicate metabolites consistently upregulated in diabetes, such as BCAAs, AAAs, DAGs, and lactate, which are associated with increased disease risk. Green arrows (↓) represent downregulated, typically protective metabolites, such as HDL, butyrate, and S1P, suggesting impaired metabolic regulation. The diagram highlights metabolic crosstalk and mechanistic interdependencies, emphasizing metabolomics-based opportunities for biomarker discovery and targeted intervention in diabetes.

As shown in **Figure 2**, branched-chain amino acids (BCAAs) and lipid intermediates play a pivotal role in metabolic reprogramming. The Framingham Heart Study established a seminal association between elevated BCAAs and aromatic amino acids (e.g., tyrosine, phenylalanine) with increased T2DM risk over a 12-year follow-up period (29). This finding has been consistently validated across multi-ethnic cohorts (70, 71), solidifying BCAAs as robust predictive biomarkers for early-stage T2DM through their demonstrated role in insulin resistance and dysregulated glucose metabolism.

Aromatic amino acids (AAAs) have similarly emerged as key metabolic regulators in diabetes progression. The METSIM study identified 3-(4-hydroxyphenyl) lactate, a phenylalanine catabolite, as significantly correlated with impaired β-cell function and elevated diabetes risk (72). Notably, ethnic-specific metabolic patterns have been observed, with tyrosine demonstrating stronger diabetogenic associations in South Asian populations compared to Europeans (73), suggesting population-specific metabolic vulnerabilities may underlie disparate T2DM incidence rates.

In addition, the imbalance of the ratio of glutamate and glutamine (Gln/Glu) is one of the key metabolic abnormalities in the development of T2DM, and its mechanism involves β-cell damage, insulin resistance, and systemic metabolic disorders. Glutamate is the main excitatory neurotransmitter in the central nervous system, which mediates neuroexcitotoxicity by activating NMDA receptors (NMDARs). Long-term NMDA treatment inhibits β-cell viability, reduces ATP synthesis, and impairs glucose-stimulated insulin secretion (GSIS) (74). Glutamine is one of the most abundant non-essential amino acids and is involved in regulating pancreatic β-cell function and insulin secretion. A case-cohort study showed that higher baseline plasma glutamate levels were associated with a significantly increased risk of T2DM, while higher glutamine levels were associated with a lower risk of T2DM, and higher Gln/Glu (glutamine-to-glutamate ratio) was associated with a significantly reduced risk of T2DM (75). This suggests that plasma glutamate and the Gln/Glu ratio can be used as predictive markers for T2DM risk.

### 3.2 Lipid metabolites

Lipid metabolism is critically involved in the pathogenesis of T2DM. Patients with T2DM often display elevated triglycerides and small, dense low-density lipoprotein (LDL) particles, as well as decreased high-density lipoprotein (HDL) cholesterol, even when their LDL cholesterol levels remain normal or near-normal (76). Certain lipid markers, such as diacylglycerols (DAGs), have been identified as early predictors of T2DM risk. Specifically, DAGs demonstrate a positive correlation with insulin resistance, as assessed by the Homeostatic Model Assessment for Insulin Resistance (HOMA-IR) (77). These findings indicate that DAGs may serve as useful biomarkers for the early detection of individuals at risk for T2DM.

A critical mediator of insulin resistance involves crosstalk between adipose tissue and hepatic metabolism, termed the “fat-liver axis” (78). Fatty acid-binding protein 4 (FABP4), an adipokine secreted by adipocytes, activates hepatic toll-like receptor 4 (TLR4) signaling, thereby promoting inflammatory responses and stimulating excessive gluconeogenesis (79). This maladaptive pathway worsens hyperglycemia and perpetuates insulin resistance. The FABP4-TLR4 signaling axis has consequently emerged as a promising therapeutic target for improving insulin sensitivity in T2DM.

Targeted metabolomics studies have further underscored the significance of acylcarnitine, key intermediates in fatty acid metabolism, as predictors of T2DM risk. Diminished mitochondrial oxidative capacity results in the accumulation of long-chain acylcarnitine, which interferes with insulin signaling pathways and impairs glucose uptake (80). These metabolites, produced during incomplete fatty acid β-oxidation, serve as sensitive biomarkers of mitochondrial dysfunction. A study has found that circulating long-chain acylcarnitine concentrations are significantly elevated in patients with T2DM (81). Elevated concentrations of specific acylcarnitine, particularly C3 and C5, correlate strongly with impaired insulin sensitivity and dysregulated glucose metabolism (66), reinforcing their potential utility in early disease detection.

### 3.3 Organic acids

Organic acids are key products of energy metabolism and intermediary metabolism (82). Alterations in their concentrations may indicate metabolic pathway dysregulation, particularly in early-stage diabetes. Variations in levels of organic acids, such as citric acid, lactic acid, and α-ketoglutaric acid, are strongly associated with insulin resistance, mitochondrial dysfunction, and impaired glucose metabolism, suggesting their potential as early diagnostic biomarkers for diabetes (83).

Citric acid, a central intermediate in the tricarboxylic acid (TCA) cycle, has demonstrated glucose-lowering effects in animal studies, significantly reducing blood glucose and insulin resistance while enhancing insulin sensitivity (84). This implies that diminished citrate levels may signal aberrant glucose metabolism and elevated diabetes risk. A clinical trial in obese, insulin-resistant children revealed that citric acid exhibited the most pronounced metabolic changes during weight loss and correlated positively with homeostasis model assessment of insulin resistance (HOMA-IR) scores (85).

α-Ketoglutarate, another pivotal TCA cycle intermediate, participates in amino acid metabolism and redox homeostasis. Depleted α-ketoglutarate levels are linked to oxidative stress and disrupted energy metabolism, potentially serving as an early marker of diabetic metabolic dysfunction (86). Blood lactate is an indicator of the gap between energy expenditure and oxidative capacity, and is clinically used to indicate energy imbalance associated with strenuous exercise, hypoxia, and ischemia (87). Insufficient oxidative capacity can lead to the development of insulin resistance and type 2 diabetes (88). Elevated fasting plasma lactate levels are observed in diabetic patients compared to non-diabetic individuals (89), with further increases noted in poorly controlled T1DM and glycogenic liver disease (90). These findings position lactate as a predictive biomarker for diabetes progression. Additionally, longitudinal lactate monitoring may aid in evaluating metabolic status and therapeutic efficacy in diabetic patients (91).

### 3.4 Bile acids

Bile acids (BAs) are not only key mediators of fat digestion and absorption, but also important signaling molecules that regulate glucose and lipid metabolism by activating nuclear receptors and membrane receptors (92). In T2DM and obesity, the synthesis, composition, and signal transduction of bile acids are significantly changed, directly involved in the process of insulin resistance (IR) and β-cell dysfunction. Bile acids are natural ligands of Farnesoid X Receptor (FXR). FXR can regulate the synthesis of bile acids itself and affect glucose metabolism (inhibit hepatic gluconeogenesis, promote glycogen synthesis, and enhance insulin sensitivity). Secondary bile acids are high-affinity ligands of TGR5. After activation, TGR5 stimulates intestinal L cells to secrete glucagon-like peptide-1 (GLP-1), promote insulin secretion, and improve blood sugar control (93).

### 3.5 Gut microbiome and its metabolic products

The gut microbiome and its metabolic products constitute another critical modulator of β-cell function. The microbial metabolite trimethylamine N-oxide (TMAO) disrupts β-cell physiology by attenuating intracellular calcium signaling, thereby compromising insulin exocytosis while simultaneously promoting endoplasmic reticulum stress and apoptotic pathways (94). TMAO concentrations are elevated in diabetic patients since TMAO can directly reduce glucose-stimulated insulin secretion (GSIS) in MIN6 cells and mouse or human pancreatic islets (95). Inhibition of TMAO production can improve β-cell GSIS, β-cell ratio, and glucose tolerance in diabetic mouse models.

In contrast, beneficial microbiota-derived short-chain fatty acids (SCFAs) like butyrate exert protective effects by suppressing pro-inflammatory cytokine signaling and maintaining normal insulin secretory capacity in β-cells (96). Compared with healthy controls, patients with type 2 diabetes have reduced intestinal flora diversity, fewer butyrate-producing bacteria, and lower fecal SCFA concentrations (97). Fecal microbiota transplantation (FMT) from healthy donors has been shown to enhance insulin sensitivity in individuals with metabolic syndrome, accompanied by increased abundance of SCFAs-producing bacteria and favorable shifts in microbial metabolites (98). These findings highlight the complex interplay between metabolic and microbial factors in β-cell dysfunction.

### 3.6 Dysregulated sphingolipid metabolism

Increasing evidence indicates that abnormal sphingolipid metabolism is deeply involved in the metabolic reprogramming of diabetes (especially T2DM) by interfering with insulin signaling, inducing chronic inflammation, destroying mitochondrial function, and promoting β-cell apoptosis (99). Mechanistically, ceramide accumulation activates pro-inflammatory signaling through the CD36/NF-κB pathway, upregulating thioredoxin-interacting protein (TXNIP) expression and inducing mitochondrial oxidative stress, which collectively impair β-cell insulin secretion and viability (100). Other sphingolipid metabolites, such as sphingosine-1-phosphate (S1P), are significantly negatively correlated with the progression of diabetes (101). S1P has anti-inflammatory and anti-apoptotic effects, and promotes β-cell survival and insulin secretion. Low S1P can be used as a biomarker to predict the severity of diabetes and provide a new target for future precision treatment.

## 4 Personalized treatment strategies in diabetes

The well-established heterogeneity of diabetes pathogenesis underscores the critical need for precision medicine approaches to achieve optimal therapeutic outcomes. Contemporary advances in high-throughput metabolomics and next-generation microbiome profiling have facilitated the discovery of novel metabolic signatures and dysregulated pathways that can inform tailored therapeutic strategies. These technological innovations enable stratification of patient populations based on distinct metabolic phenotypes, thereby paving the way for truly personalized diabetes management.

### 4.1 Drug response stratification

Patient stratification based on pharmacological response represents a cornerstone of personalized diabetes management, particularly for metformin as first-line therapy in T2DM. Despite its clinical ubiquity, metformin demonstrates marked interpatient variability in therapeutic efficacy (102). Approximately 30% of patients failing to achieve adequate glycemic control on monotherapy (103), highlights the imperative to elucidate the biological determinants of treatment heterogeneity.

Emerging evidence implicates the gut microbiome as a key modulator of metformin pharmacodynamics. Mechanistic studies reveal that microbial processing of glutamine and related amino acids, along with purine metabolism byproducts, may condition metformin activity through microbiome-mediated metabolic reprogramming (104). Specific commensal organisms enhance therapeutic efficacy via production of guanidine derivatives (e.g., agmatine) that synergize with metformin to suppress hepatic gluconeogenesis and modulate intestinal glucose absorption. Clinically, patients possessing microbiota with enriched biosynthetic capacity for these metabolites exhibit superior glycemic responses, positioning microbiome profiling as a promising predictive biomarker for treatment stratification. Concurrently, pharmacogenomic investigations have identified functional polymorphisms in metformin transporter genes as critical determinants of interindividual variability. Single-nucleotide polymorphisms (SNPs) in organic cation transporters (OCT1/SLC22A1, OCT2/SLC22A2, OCT3/SLC22A3) significantly impact drug pharmacokinetics through altered intestinal absorption, hepatic distribution, and renal clearance (105). Notably, the rs622342 (A>C) and rs72552763 (C>A) variants in SLC22A1 correlate with differential HbA1c reduction and plasma drug concentrations. Complementary evidence links OCT2/OCT3 regulatory SNPs (rs7757336, rs2481030) to impaired bioavailability and diminished therapeutic response, as demonstrated through integrated pharmacogenetic and pharmacokinetic analyses (106).

Therapeutic response to glucagon-like peptide-1 (GLP-1) receptor agonists exhibits significant interindividual variability that can be stratified using metabolomic profiling. Among emerging biomarkers, lysophosphatidic acid (LPA) - a bioactive phospholipid with pro-inflammatory properties - has been identified as a clinically relevant predictor of pharmacological response. Mechanistically, LPA contributes to insulin resistance through dual pathways: (1) activation of inflammatory cascades via LPAR1–6 receptors, and (2) direct impairment of insulin receptor substrate (IRS) phosphorylation. Elevated circulating LPA concentrations may attenuate GLP-1 efficacy by disrupting receptor-mediated insulinotropic signaling pathways (107). Beyond its glucoregulatory effects, liraglutide demonstrates significant antioxidant properties as evidenced by clinical trial data. Treatment with liraglutide results in substantial reductions in established oxidative stress markers, including thiobarbituric acid reactive substances (TBARS) and malondialdehyde (MDA), particularly in patients exhibiting favorable baseline antioxidant capacity as measured by superoxide dismutase (SOD) and glutathione peroxidase (GPx) activity (108). These findings suggest that pretreatment antioxidant status may serve as a predictive biomarker for liraglutide’s pleiotropic effects.

In addition, fibroblast growth factor 21 (FGF21) analogs exhibit variable therapeutic efficacy that correlates with baseline metabolic profiles. Preclinical and early-phase clinical studies demonstrate that patients with elevated BCAAs and reduced adiponectin concentrations show attenuated responses to FGF21 therapy, likely reflecting impaired metabolic flexibility characterized by mitochondrial dysfunction and adipose tissue inflammation. These metabolic signatures may serve as stratification biomarkers for FGF21 treatment selection.

Similarly, response heterogeneity to sodium-glucose cotransporter-2 (SGLT2) inhibitors is associated with distinct pre-treatment metabolic states. Metabolomic analyses reveal that patients with lower circulating 3-hydroxyisobutyric acid (3-HIB) - a valine catabolite indicative of mitochondrial stress - achieve superior glycemic control and cardiovascular risk reduction following SGLT2 inhibition. This association positions 3-HIB as both a pathophysiological marker of metabolic inflexibility and a potential predictor of therapeutic response, with mechanistic studies suggesting its role in impairing endothelial function and promoting cardiac lipotoxicity.

### 4.2 Optimization of nutritional interventions

Nutritional strategies are integral to diabetes management. Time-restricted eating (TRE), for example, has been shown to reprogram hepatic lipid metabolism by aligning feeding patterns with circadian rhythms (109). TRE reduces hepatic steatosis and improves insulin sensitivity by enhancing the expression of lipid-oxidizing enzymes during fasting periods. This approach leverages metabolic rhythms to maximize therapeutic benefits. Prebiotics, such as inulin, modulate gut microbiota by promoting the growth of beneficial bacteria (e.g., Bifidobacteria), which may enhance butyrate production and exert anti-inflammatory effects. Clinical trials demonstrate that inulin supplementation improves insulin sensitivity in overweight individuals, likely through these microbiota-mediated mechanisms (110).

Nutritional strategies can be further optimized based on individual metabolic responses. For instance, omega-3 can prevent and reverse insulin resistance (IR) induced by a high-fat diet, improve glucose metabolism in patients with prediabetes (such as obese children or adults with metabolic syndrome), reduce systemic inflammation, and enhance metabolic flexibility (111). Omega-3 fatty acids competitively inhibit the cyclooxygenase (COX) and lipoxygenase (LOX) pathways, reduce the production of pro-inflammatory eicosanoids (such as prostaglandins and leukotrienes), and the pro-resolution mediators derived from them (such as resolving and protecting) can actively relieve inflammation. Additionally, foods rich in polyphenols (such as coffee, green tea, red wine, olive oil, cocoa, etc.) can reduce fasting blood glucose and HbA1c, reduce insulin resistance, and inhibit inflammation and oxidative stress. In clinical trials, they have shown the potential to improve blood glucose metabolism, insulin sensitivity, and vascular function in patients with T2DM (112).

## 5 Technical challenges and cutting-edge breakthroughs

### 5.1 Existing technical bottlenecks

#### 5.1.1 Technical limitations in sampling and pre-analytical handling

Temporal standardization of biological sampling represents a fundamental challenge in diabetes metabolomics research (113). Metabolite concentrations exhibit substantial diurnal variation and physiological state-dependent fluctuations, particularly between fasting and postprandial conditions. Li et al. demonstrated that postprandial concentrations of BCAAs (valine, leucine, isoleucine) and short-chain acylcarnitine (C3-C5) undergo significant changes, with diabetes patients showing particularly pronounced postprandial increases in specific metabolites (e.g., C16:1 acylcarnitine concentrations rising by 30% compared to fasting levels) (114). Such dynamic variations may obscure genuine metabolic signatures, potentially yielding false-positive associations or masking fasting-state pathophysiological patterns.

Pre-analytical factors such as anticoagulant type, centrifugation protocol, and storage temperature also significantly impact metabolite stability and measurement accuracy. Jobard et al. showed that EDTA versus heparin selection leads to measurable differences in amino acid concentrations, while prolonged room temperature storage degrades short-chain fatty acids and nucleotides, introducing technical bias into metabolic profiling (115).

#### 5.1.2 Biological confounders in metabolomic profiling

In parallel, biological variables contribute additional layers of complexity to diabetes metabolomics. These include diet, physical activity, medication use, and comorbidities, each of which can significantly reshape the metabolic landscape. A systematic review by Kim demonstrated that metformin treatment alters the plasma and urine metabolomes by modulating gut microbiota and energy metabolism, while high-fat diets further exacerbate metabolic imbalances (116). These biological factors, if unaccounted for, may confound disease-specific metabolic signatures and complicate downstream analyses.

#### 5.1.3 Pharmacologic and demographic sources of variation

Disease states (such as cancer or diabetes) themselves can also lead to significant changes in branched-chain amino acid and lipid metabolites. In the context of diabetes, pharmacological interventions can modulate metabolic pathways independently of disease mechanisms. For example, metformin therapy significantly modifies serum phosphatidylcholine profiles (notably acyl-alkyl PC species) in T2DM (117), potentially confounding the interpretation of insulin resistance-associated metabolic signatures.

Population-scale metabolomic studies must additionally account for substantial interindividual variability stemming from demographic, anthropometric (e.g., BMI effects), and lifestyle factors that collectively influence baseline metabolic set points. The inherent biological complexity of metabolites, functioning not merely as passive biomarkers but as dynamic regulators interacting with genomic, epigenetic, and proteomic networks (118), poses unique challenges for establishing clinically relevant reference ranges. This complexity is compounded by the multitude of influencing factors, including nutritional status, hydration state, and physical activity patterns, necessitating rigorous standardization protocols for meaningful metabolomic investigation in diabetes research.

#### 5.1.4 Metabolite annotation bottlenecks

Accurately annotating unknown metabolites remains one of the most persistent and complex challenges in metabolomics research. Despite continuous advancements in analytical technology, including high-resolution mass spectrometry, only approximately 10% of detected metabolites can be identified through mass spectral library matching, leaving the remaining 90% unknown (119). These unidentified signals often correspond to low-abundance or novel metabolites that may serve as critical biomarkers, particularly in diseases such as diabetes (120). However, the absence of comprehensive reference libraries, spectral databases, and standardized annotation pipelines hinders their biological interpretation (121). For example, Tan et al. identified only 1,088 endogenous compounds using high-resolution mass spectrometry, while a significant number of lipids remain insufficiently annotated due to structural complexity and limited spectral coverage, potentially introducing bias in downstream pathway analyses (122). This issue is especially pronounced for lipid subclasses such as ceramides, which play a crucial role in driving insulin resistance via the Akt signaling pathway but are challenging to interpret due to their structural heterogeneity and inadequate mass spectrometric coverage (123).

#### 5.1.5 Data preprocessing and quality control

Furthermore, data preprocessing remains a major technical bottleneck due to the high dimensionality and inherent variability of metabolomic datasets. Key preprocessing steps such as peak filtering, imputation of missing values, and normalization can profoundly influence analytical outcomes (124). Even when standardized acquisition protocols are employed, technical variation can obscure true biological signals. Abiotic factors, including instrumental errors, can contribute substantially to variability in untargeted metabolomics studies, necessitating rigorous quality control measures (125).

### 5.2 Innovative technological breakthroughs

#### 5.2.1 Metabolic flux analysis and stable isotope tracing

Dynamic metabolic flux analysis, particularly utilizing 13C-labeled glucose tracers, has become an indispensable technique for investigating hepatic gluconeogenesis in diabetes. Unlike traditional static metabolomics, which provides only a snapshot of metabolite concentrations, this approach allows for real-time quantification of carbon flux through specific metabolic pathways, offering dynamic insights into altered hepatic metabolism. For instance, a study employed global 13C tracing and non-targeted mass spectrometry to analyze intact human liver tissue ex vivo (126). This research provided a comprehensive overview of hepatic metabolic fluxes, enhancing our understanding of liver metabolism in the context of metabolic diseases, including diabetes. Furthermore, a study utilized multi-tissue 2H/13C flux analysis to reveal compensatory upregulation of renal gluconeogenesis in hepatic PEPCK-C-knockout mice, highlighting the complexity of gluconeogenic regulation in diabetic conditions (127). 13C magnetic resonance spectroscopy (13C-MRS) has emerged as a powerful noninvasive tool for quantifying hepatic mitochondrial oxidative and anaplerotic fluxes in humans. Befroy et al. applied dynamic 13C-MRS to healthy individuals, demonstrating the feasibility of directly measuring hepatic TCA cycle and pyruvate carboxylase–mediated cataplerotic fluxes *in vivo*, providing a methodological framework for studying hepatic gluconeogenesis in metabolic disorders (128). Similarly, as demonstrated by Hasenour et al., the choice of isotopic tracers (e.g., 13C-propionate vs. 13C-lactate) and modeling assumptions significantly impact hepatic flux estimates, highlighting the need for rigorous experimental design in metabolic studies (129). Moreover, Kim et al. provided a detailed methodological framework for integrating 13C-based metabolic flux analysis (MFA) in metabolic studies (130). They emphasized that precise control of infusion protocols, compartmental modeling, and integration of multi-precursor data are crucial for accurately quantifying pathways such as glycogenolysis and gluconeogenesis. 13C-MFA has been used to track hepatic TCA cycle fluxes and gluconeogenesis, offering insights into metabolic dysregulation in diabetes-associated conditions. Young et al. summarized how *in vivo* 13C and 2H tracing can capture whole-body flux alterations, including the role of lactate as a key carbon shuttle between tissues (131). However, the relative contribution of recycled lactate to diabetic hyperglycemia remains an active area of research.

#### 5.2.2 Artificial intelligence for predictive metabolomics

The integration of artificial intelligence (AI), particularly deep learning algorithms, has profoundly expanded the analytical capacity of metabolomics in diabetes research. Unlike conventional statistical methods, AI models can efficiently process high-dimensional, nonlinear, and heterogeneous metabolomics data, thereby uncovering hidden associations between metabolic profiles and disease phenotypes. A recent study demonstrated that combining untargeted plasma metabolomics with machine learning algorithms can effectively identify metabolite signatures predictive of glycemic deterioration and progression from prediabetes to T2DM (58). This approach not only enhanced risk stratification beyond classical clinical markers but also revealed novel metabolic features with potential relevance as therapeutic targets, many of which were not captured through traditional enrichment or univariate analyses. A notable example of clinical application is the study by Huang et al., who used machine learning approaches to identify metabolic signatures of incident chronic kidney disease (CKD) in individuals with prediabetes and type 2 diabetes (132). Through targeted metabolomics and machine learning methods, the study identified sphingomyelin C18:1 and phosphatidylcholine diacyl C38:0 as candidate metabolite biomarkers of incident CKD specifically in hyperglycemic individuals. The developed prediction models outperformed the currently established clinical algorithm for CKD, demonstrating the power of machine learning in improving risk prediction for diabetes-related complications. Additionally, based on the Bagged CART integrated algorithm and metabolomics features, diabetic retinopathy subtypes (NDR/NPDR/PDR) can be effectively classified with an accuracy of 72% and a sensitivity of 91.9%, proving that artificial intelligence models can assist in the early detection and accurate classification of diabetic complications. A convolutional neural network (CNN)-based deep learning model trained on untargeted plasma metabolomics could accurately identify predictive metabolite signatures linked to glycemic control and complications in type 2 diabetes patients. The model also suggested potential drug targets and stratification markers that were not apparent through classical pathway enrichment analysis. As described by Kirtipal et al., by integrating intestinal metagenome, metabolome, and host data, machine learning algorithms can predict individual glycemic responses and reveal that microbiota metabolites (such as SCFAs) affect diabetes progression by regulating insulin sensitivity, providing a new strategy for precision nutrition (133). Based on the Bagged CART integrated algorithm and metabolomics features, diabetic retinopathy subtypes (NDR/NPDR/PDR) can be effectively classified with an accuracy of 72% and a sensitivity of 91.9%, proving that artificial intelligence models can assist in the early detection and accurate classification of diabetic complications (134).

Despite these advancements, AI-driven metabolomics models still face challenges in reproducibility and interpretability. Differences in data preprocessing and cohort characteristics can affect model stability, while the “black box” nature of deep learning algorithms limits clinical acceptance. Incorporating interpretability tools such as Shapley Additive Explanations (SHAP) and ensuring cross-cohort validation are essential steps for clinical translation (135).

## 6 Clinical transformation and prospects

### 6.1 Diagnostic reagent development

Especially in the early diagnosis and risk assessment of diabetes, metabolomics technology has shown great potential in the field of diagnostic reagent development, and its application has gradually entered the clinical transformation stage. In recent years, the development of diagnostic reagents based on metabolomics has made significant progress, among which NMR and LC-MS technologies have become mainstream tools. For instance, a study utilizing ultra-high-performance liquid chromatography coupled with tandem mass spectrometry (UHPLC-MS/MS) analyzed serum samples to identify metabolites associated with diabetes risk (136). This targeted metabolomics approach demonstrated the potential to detect metabolic alterations preceding diabetes onset, suggesting its utility in early diagnosis and risk assessment. Furthermore, UHPLC-MS/MS has been employed to quantify amino acids and acylcarnitine in plasma, providing insights into metabolic disturbances linked to insulin resistance and diabetes (137). Such analyses contribute to the development of diagnostic tools aimed at early intervention strategies for at-risk individuals. In addition to UHPLC-MS/MS, other advanced mass spectrometry techniques have been instrumental in diabetes research. For example, LC-MS/MS and GC-MS have significantly broadened the spectrum of detectable metabolites, even at lower concentrations (138). These technologies have enabled the identification and quantification of potential biomarkers associated with diabetes and its complications, providing new avenues for clinical diagnostics and metabolic studies.

With the advancement of technology, the platform for metabolomics detection has gradually moved from large laboratories to clinical sites. The development of microfluidic chip technology provides new possibilities for point-of-care testing (POCT) of metabolomics. Microfluidic chips can achieve rapid analysis of complex metabolites in small devices by integrating sample processing, separation, and detection functions (139). In recent years, the coupling of microfluidic chips and mass spectrometry detection has become a research hotspot, greatly improving the sensitivity and efficiency of detection. The research team has developed a metabolomics detection platform based on microfluidic chips, which can separate 16 amino acids in less than two minutes without extensive sample preparation (140). This device is not only portable, but also can monitor the metabolic status of patients in real time, providing clinicians with a rapid basis for diagnosis. In the future, with the further optimization of microfluidic technology, metabolomics detection is expected to play a greater role in family medical care and community health management.

However, the development and clinical application of metabolomics diagnostic reagents still face some challenges. First, the biomarker combination of metabolite profiles needs to be further verified for its clinical value. At present, although metabolomics has identified numerous disease-associated metabolites, their translational potential requires further validation in diverse clinical cohorts (118). Despite the growing literature on metabolomics in diabetes, a comparative understanding of cohort designs, biomarker patterns, and analytical strategies remains limited. To provide a synthesized perspective, we compiled a representative set of metabolomics studies (**Table 2**) that have advanced the field toward clinical translation. These studies vary in sample size, biospecimen types, and analytical platforms (e.g., LC-MS, NMR, GC-MS), yet converge on key biomarker trends such as elevated BCAAs, ceramides, and α-hydroxybutyrate in diabetic or insulin-resistant populations.

**Table 2:** Representative metabolomics studies highlighting key biomarkers and platforms for diabetes risk prediction.

Study	Cohort/Sample Size	Biofluid	Platform	Key Metabolites	Clinical Application
Wang et al. (2011) (29)	Framingham: 189 T2D + 189 controls	Plasma	LC-MS/MS	BCAAs, Aromatic AAs	Prediction of future T2D risk
Floegel et al. (2013) (141)	EPIC-Potsdam: 849 incident T2D cases KORA: 876 participants, 91 incident T2D cases TüF: 76 individuals	Serum (EPIC/KORA) Plasma (TüF)	FIA-MS/MS	Hexose, Phenylalanine, Phosphatidylcholines	Improves T2D risk prediction
Wang-Sattler et al. (2012) (81)	KORA Cohort: 4,261 participants in S4 (cross-sectional) and 1,010 in F4 (prospective).	Serum	LC-MS/MS	Glycine, LPC (18:2), Acetylcarnitine	Early T2D prediction and risk stratification
Yu et al. (2015) (142)	976 Chinese adults (40–74 years)	Plasma	UHPLC & GC-MS	Hexoses, Valine, 3-Methoxytyrosine	Improves T2D risk prediction in Chinese populations
Menni et al. (2013) (143)	TwinsUK Cohort: 2,204 females 115 T2D cases, 192 IFG, 1,897 controls	Plasma	Metabolon Nontargeted Metabolomics	3-Methyl-2-oxovalerate, BCAAs	Early detection of insulin resistance (IFG)
Papandreou et al. (2019) (144)	PREDIMED: 700 participants	Plasma	LC-MS/MS	Isoleucine, Alanine, Glycine	Improves prediction of insulin resistance (HOMA-IR) and future T2D risk
Liu et al. (2025) (145)	UK Biobank: 98,831 participants	Plasma	NMR (168 metabolites)	Triglycerides, HDL, BCAAs	T2DM risk stratification and prediction

Secondly, the technical standardization and quality control of the detection platform are the key to ensuring the reliability of the test results (146). The U.S. Food and Drug Administration (FDA) has set strict requirements for the validation of biomarkers, including sensitivity, specificity, and repeatability (147). In addition, the analysis and interpretation of metabolomics data also require more intelligent tools. In recent years, machine learning-based metabolomics data analysis methods have gradually emerged, which can mine potential biomarker combinations from massive data and predict the risk and prognosis of diseases (148). A recent study developed a deep learning framework trained on plasma metabolite spectra to predict the risk of progression from prediabetes to T2DM. This model identified a panel of nine circulating metabolites that significantly improved predictive performance and provided a robust basis for early identification and intervention in high-risk individuals (58).

In the future, the development of metabolomics diagnostic reagents will move towards high precision, instant, and intelligent directions. On the one hand, with the continuous advancement of detection technology, the sensitivity and throughput of metabolomics detection will be further improved, which can more comprehensively reflect the metabolic status of patients. On the other hand, the popularization of POCT equipment will make metabolomics detection more convenient and enable rapid transformation from laboratory to the clinical site. The introduction of artificial intelligence technology will greatly improve the analysis ability of metabolomics data and provide more accurate support for diagnosis and personalized treatment. Overall, the development of metabolomics diagnostic reagents will not only promote the early diagnosis and precise treatment of diabetes but also provide new tools and methods for the clinical management of other metabolic diseases.

### 6.2 Therapeutic target mining

Metabolomics-driven analyses have increasingly facilitated the discovery of novel therapeutic targets in diabetes. For instance, targeted lipidomic profiling has identified ceramide species as correlates of insulin resistance, which led to the investigation of CerS6 as a driver of mitochondrial dysfunction and adipose inflammation (149). Similarly, metabolite profiling of bile acid derivatives highlighted TGR5 as a key receptor modulating GLP-1 secretion and energy expenditure (150). Metabolomics has also supported the identification of FFAR2, a short-chain fatty acid receptor, through correlation with plasma SCFA levels and metabolic phenotypes (151, 152). These findings underscore how metabolomics can inform precision pharmacology by linking endogenous metabolic shifts with actionable molecular targets.

### 6.3 Future development directions

The future development direction of metabolomics technology will focus on three core areas: multi-omics integration, real-time monitoring technology, and ethics and norms, to promote the application of metabolomics in precision medicine and personalized medicine.

Combining metabolomics with other omics data, such as genomics, proteomics, and transcriptomics, offers a comprehensive understanding of diabetes pathophysiology. This integrative approach is particularly powerful for dissecting the molecular basis of diabetic complications and for identifying context-specific biomarkers across tissues and comorbid conditions (153). It also facilitates the discovery of novel biomarkers and therapeutic targets. For instance, a multi-omics study integrating epigenomics, whole-genome sequencing, and metabolomics uncovered previously unrecognized pathways implicated in T2DM, underscoring the value of systems-level strategies in unraveling complex disease mechanisms (154). At the same time, AI and ML algorithms are increasingly applied to metabolomics data to predict disease progression and personalize treatment strategies. For example, integrating ML approaches with metabolomics panels has improved the prediction of T2DM, enabling early intervention (155). A recent study employed ML models on quantitative metabolomics data to accurately predict the 4-year risk of developing T2DM, demonstrating the strong potential of metabolomics-based models for early detection and prevention (156). In addition, a study in Diabetes integrated ML techniques with metabolomics to identify metabolic signatures predictive of CKD in individuals with T2DM or prediabetes, enabling early identification of high-risk individuals for timely intervention (132).

The development of real-time monitoring technology will promote metabolomics from the laboratory to clinical application, and the development of wearable devices that can monitor metabolic parameters in real time is changing diabetes management (157). As non-invasive techniques for monitoring metabolic changes gain traction, innovations such as a sweat-based wearable sensor show potential for monitoring blood sugar levels and other metabolites without the need for invasive surgery (158). In addition, integrating CGM data with other wearable technology, such as activity monitors, has been shown to improve metabolic outcomes in individuals with T2DM (157). Furthermore, advancements in wearable insulin biosensors that combine real-time glucose monitoring with automated insulin delivery are enhancing glycemic control and quality of life for diabetes patients (159). These innovations underscore the potential of wearable technology to revolutionize personalized diabetes care.

The clinical implementation of metabolomics requires careful consideration of data privacy, ethical standards, and regulatory compliance. Given the sensitivity of metabolomics data, especially when collected longitudinally, it is imperative that informed consent protocols explicitly address critical issues such as data reuse, participant re-identifiability, and the integration of data across multiple platforms. Researchers should ensure that participants are fully informed about the extent of data use, how their data will be processed, and any potential risks associated with cross-platform integration (160). In addition, researchers should consider the adoption of robust data security measures, such as encryption and de-identification, to protect the privacy of participants’ data. The use of anonymized datasets, stored in secure, open-access repositories, should be encouraged to facilitate data sharing and enhance reproducibility, while ensuring privacy and compliance with data protection laws. Furthermore, data governance should be aligned with international standards, such as the General Data Protection Regulation (GDPR) in Europe and similar regulations in other regions, to ensure the ethical and legal handling of metabolomics data. To facilitate ethical translation, researchers are encouraged to adopt open-access repositories with anonymized datasets and align data governance with international standards such as the General Data Protection Regulation (GDPR) (161). To further support clinical and translational applications, researchers should engage with established frameworks, such as those provided by the Metabolomics Standards Initiative (MSI), which promote the harmonization of reporting, data quality, and traceability (162). These guidelines offer valuable resources for maintaining data integrity, ensuring consistency across studies, and improving the overall credibility of metabolomics research. By adopting these ethical and regulatory practices, researchers can help ensure the responsible and impactful use of metabolomics in clinical settings.

## 7 Conclusion

Metabolomics has emerged as a transformative pillar in deciphering the complex metabolic landscape of diabetes, offering unprecedented access to real-time biochemical states that underlie disease onset, progression, and therapeutic response. In contrast to traditional diagnostic approaches that typically capture static or downstream manifestations of disease, metabolomics enables dynamic profiling of metabolic fluxes, revealing subtle yet clinically meaningful perturbations that precede overt hyperglycemia or complications. By detecting disease-relevant metabolites, such as branched-chain amino acids, lipid intermediates, bile acids, and gut microbiota–derived compounds, metabolomics facilitates early risk prediction, mechanistic stratification, and precision-guided interventions.

Moreover, this systems-level perspective on metabolic reprogramming has deepened our understanding of heterogeneous disease trajectories, including subtypes of insulin resistance, patterns of β-cell failure, and variations in drug responsiveness. As emphasized in this review, the integration of advanced computational tools, particularly machine learning and artificial intelligence, has greatly enhanced the analytical power of metabolomics, enabling the extraction of predictive biomarker panels and individualized treatment profiles from high-dimensional datasets. Likewise, the convergence of metabolomics with other omics layers, such as transcriptomics, genomics, and macrobiomics, marks a new era of integrated systems biology in diabetes research.

Nevertheless, the translation of metabolomic discoveries into clinical practice remains incomplete. Key bottlenecks, including the lack of standardized protocols, limited inter-laboratory harmonization, insufficient regulatory frameworks for biomarker validation, and challenges in biological interpretation, must be systematically addressed. In parallel, ethical considerations surrounding data privacy, informed consent, and the clinical implementation of AI-driven metabolic models must be carefully navigated.

Looking ahead, advances in point-of-care metabolomics platforms, wearable biosensors, and minimally invasive diagnostics may revolutionize diabetes management by enabling continuous metabolic monitoring and real-time clinical decision-making. Realizing this vision will require sustained interdisciplinary collaboration, regulatory foresight, and a strong commitment to patient-centered innovation. By meeting these imperatives, metabolomics is well-positioned to redefine diabetes diagnostics and usher in a new era of predictive, preventive, and personalized metabolic medicine.
